# Ex vivo observation of granulocyte activity during thrombus formation

**DOI:** 10.1186/s12915-022-01238-x

**Published:** 2022-02-07

**Authors:** Daria S. Morozova, Alexey A. Martyanov, Sergei I. Obydennyi, Julia-Jessica D. Korobkin, Alexey V. Sokolov, Ekaterina V. Shamova, Irina V. Gorudko, Anna L. Khoreva, Anna Shcherbina, Mikhail A. Panteleev, Anastasia N. Sveshnikova

**Affiliations:** 1 Pirogov Russian National Research Medical University, Ostrovityanova Str., 1, Moscow, 117997 Russia; 2 National Medical Research Centre of Pediatric Hematology, Oncology and Immunology named after Dmitry Rogachev, 1 Samory Mashela St, Moscow, 117198 Russia; 3 Center for Theoretical Problems of Physicochemical Pharmacology, Russian Academy of Sciences, 30, Srednyaya Kalitnikovskaya str., Moscow, 109029 Russia; 4 Institute for Experimental Medicine, 12 Acad. Pavlova str., Saint Petersburg, 197376 Russia; 5 Department of Biophysics, Faculty of Physics, Belarusian State University, 220030 Minsk, Belarus; 6 Faculty of Physics, Lomonosov Moscow State University, 1/2 Leninskie Gory, Moscow, 119991 Russia; 7 Department of Normal Physiology, Sechenov First Moscow State Medical University, 8/2 Trubetskaya St., Moscow, 119991 Russia

**Keywords:** Thromboinflammation, Platelets, Granulocytes, Chemotaxis, Flow chambers

## Abstract

**Background:**

The process of thrombus formation is thought to involve interactions between platelets and leukocytes. Leukocyte incorporation into growing thrombi has been well established in vivo, and a number of properties of platelet-leukocyte interactions critical for thrombus formation have been characterized in vitro in thromboinflammatory settings and have clinical relevance. Leukocyte activity can be impaired in distinct hereditary and acquired disorders of immunological nature, among which is Wiskott-Aldrich Syndrome (WAS). However, a more quantitative characterization of leukocyte behavior in thromboinflammatory conditions has been hampered by lack of approaches for its study ex vivo. Here, we aimed to develop an ex vivo model of thromboinflammation, and compared granulocyte behavior of WAS patients and healthy donors.

**Results:**

Thrombus formation in anticoagulated whole blood from healthy volunteers and patients was visualized by fluorescent microscopy in parallel-plate flow chambers with fibrillar collagen type I coverslips. Moving granulocytes were observed in hirudinated or sodium citrate-recalcified blood under low wall shear rate conditions (100 s^−1^). These cells crawled around thrombi in a step-wise manner with an average velocity of 90–120 nm/s. Pre-incubation of blood with granulocyte priming agents lead to a significant decrease in mean-velocity of the cells and increase in the number of adherent cells. The leukocytes from patients with WAS demonstrated a 1.5-fold lower mean velocity, in line with their impaired actin polymerization. It is noteworthy that in an experimental setting where patients’ platelets were replaced with healthy donor’s platelets the granulocytes’ crawling velocity did not change, thus proving that WASP (WAS protein) deficiency causes disruption of granulocytes’ behavior. Thereby, the observed features of granulocytes crawling are consistent with the neutrophil chemotaxis phenomenon. As most of the crawling granulocytes carried procoagulant platelets teared from thrombi, we propose that the role of granulocytes in thrombus formation is that of platelet scavengers.

**Conclusions:**

We have developed an ex vivo experimental model applicable for observation of granulocyte activity in thrombus formation. Using the proposed setting, we observed a reduction of motility of granulocytes of patients with WAS. We suggest that our ex vivo approach should be useful both for basic and for clinical research.

**Supplementary Information:**

The online version contains supplementary material available at 10.1186/s12915-022-01238-x.

## Background

A complex interplay between blood coagulation system, immune system, and endothelium, called thromboinflammation, occurs in diverse pathophysiological situations, such as bacterial infection or cancer [1]. Thromboinflammation is thought to be driven mainly by the interactions between granulocytes and platelets [2]. Platelets are non-nuclear cells, produced by the megakaryocytes, that have multiple functions both in hemostasis and immunity [3, 4]. Platelet activation at the site of injury or inflammation leads to the secretion of platelet α-granules, which contain P-selectin, fibrinogen, VWF, growth factors, and chemoattractants for leukocytes (NAP2, RANTES, CD40L, etc.) [5, 6]. These proteins play a crucial role in the leukocyte recruitment and adhesion [7–9]. The adhesion to platelets causes leukocytes’ integrins activation [10], and their migration through thrombi [11]. Therefore, platelet-leukocyte interactions are in the heart of thromboinflammation.

Granulocytes’ migration in thrombi should be dependent on concentrations of their chemoattractants. The contact with a chemoattractant (“priming”) of granulocyte leads to their β2-integrins (CD11a/CD18, LFA-1 (αLβ2) and mainly CD11b/CD18, Mac-1 (αMβ2)) activation, which results in their firm adhesion to the surface. Therefore, we expect that granulocyte movement in a thrombus should be influenced by their priming. An inhibition of actin polymerization impairs granulocyte chemotaxis [12]. In patients with cytoskeletal abnormalities, for instance, with Wiskott-Aldrich syndrome (genetic hemorrhagic and immunological syndrome; WAS) [13, 14], we expect impaired granulocyte involvement in the thrombus formation process.

The study was aimed at the development and validation of an ex vivo technique, allowing observation and simulation of the thrombus-leukocyte interactions (thrombus growth and leukocyte activity). To validate the method, we loaded the hirudin-anticoagulated whole blood of healthy donors or patients with WAS in parallel-plate flow chambers under the low flow shear rate (100 s^−1^). The samples from healthy donors were studied under control conditions as well as with leukocyte-priming reagents. We have identified conditions for granulocytes’ observation and derived a plethora of parameters for granulocytes’ characterization. These parameters were used for the analysis of the whole blood of patients with WAS.

## Results

### Granulocytes crawl among the growing thrombi under low wall shear rate conditions

Parallel plate flow chambers are a widely applied modern tool for the hemostasis assessment [15]. We used this tool for the assessment of the leukocyte incorporation into thrombus formation (Additional file 1: Fig. S1; Additional file 2: Video). For this purpose, we used five most known anticoagulants: EDTA and citrate (both chelate calcium), heparin and hirudin (inhibit thrombin indirectly and directly, correspondingly) [16]. For the identification of nuclear cells (NCs), blood was loaded with Hoechst 33342 (intracellular DNA), while DiOC6 (membrane potential) was used to visualize both NCs and other cell types (such as platelets). No adherent NCs were observed in EDTA or citrate anticoagulated blood (Additional file 1: Fig. S1A-H). Upon calcium replenishment to the physiological concentration in the citrated blood (Additional file 1: Fig. S1I-L) as well as in heparin or hirudin anticoagulated blood (Additional file 1: Fig. S1M-T), NCs crawling among the thrombi was observed (Additional file 1: Fig. S1A-C).

We observed rolling or crawling NCs among the thrombi, growing in the flow chamber on fibrillar collagen, as well as some motionless ones (Additional file 3: Video). The cells’ instant velocity changed from 0.014 to 0.21 μm/s and the mean velocity was 0.116 ± 0.017 μm/s (Additional file 1: Fig. S2B, D, F, Table 1). Detailed comparison between the anticoagulant impact on NCs behavior is given in the Table 1. Although the citrate recalcification setting allows observation of both plasma and platelet hemostasis, in a large fraction of experiments, the flow chamber becomes occluded by the growing thrombi (data not shown). To avoid it, we added 50 ATU (12.5% from the standard anticoagulation) of hirudin upon recalcification. Compared to hirudin, in the heparin-anticoagulated blood more NCs attached to the surface (Additional file 1: Fig. S3A), while the NCs trajectories were shorter and the number of motionless NCs was nonsignificantly larger (Additional file 1: Fig. S3B, Table 1). This attests that the NCs are at least primed by heparin, in line with previous data [17, 18]. Thus, hirudin was used as anticoagulant in all further experiments.

**Table 1:** NC behavior after 30 min of blood perfusion. Data for *N* = 5 donors

	EDTA	Citrate	Citrate recalcified	Heparin	Hirudin
NC velocity, μm/s					
Minimal instant	0.072 ± 0.022	0.07 ± 0.06	0.022 ± 0.003	0.018 ± 0.005	0.022 ± 0.017
Maximal instant	0.65 ± 0.14	0.58 ± 0.14	0.62 ± 0.03	0.567 ± 0.07	0.703 ± 0.006
Average	0.30 ± 0.09	0.32 ± 0.07	0.137 ± 0.018	0.095 ± 0.012	0.116 ± 0.017
NС trajectory length, μm					
Minimal	30 ± 21	24 ± 11	30 ± 7	23 ± 9	26 ± 15
Maximal	221 ± 69	228 ± 40	361 ± 114	400 ± 67	469 ± 25
Median	82 ± 46	65 ± 35	148 ± 62	137 ± 9	216 ± 44
Motionless NCs (velocity < 0.045 μm/s), %	–	–	25 ± 13	15 ± 5	7 ± 4
Number of attached NCs per FOV	–	–	0.52 ± 0.23	1.6 ± 0.6	1.1 ± 0.6
Thrombus area, % of FOV	3.6 ± 2.3	21 ± 3	19.9 ± 2.2	9.7 ± 0.9	12 ± 4

**Fig. 1:**
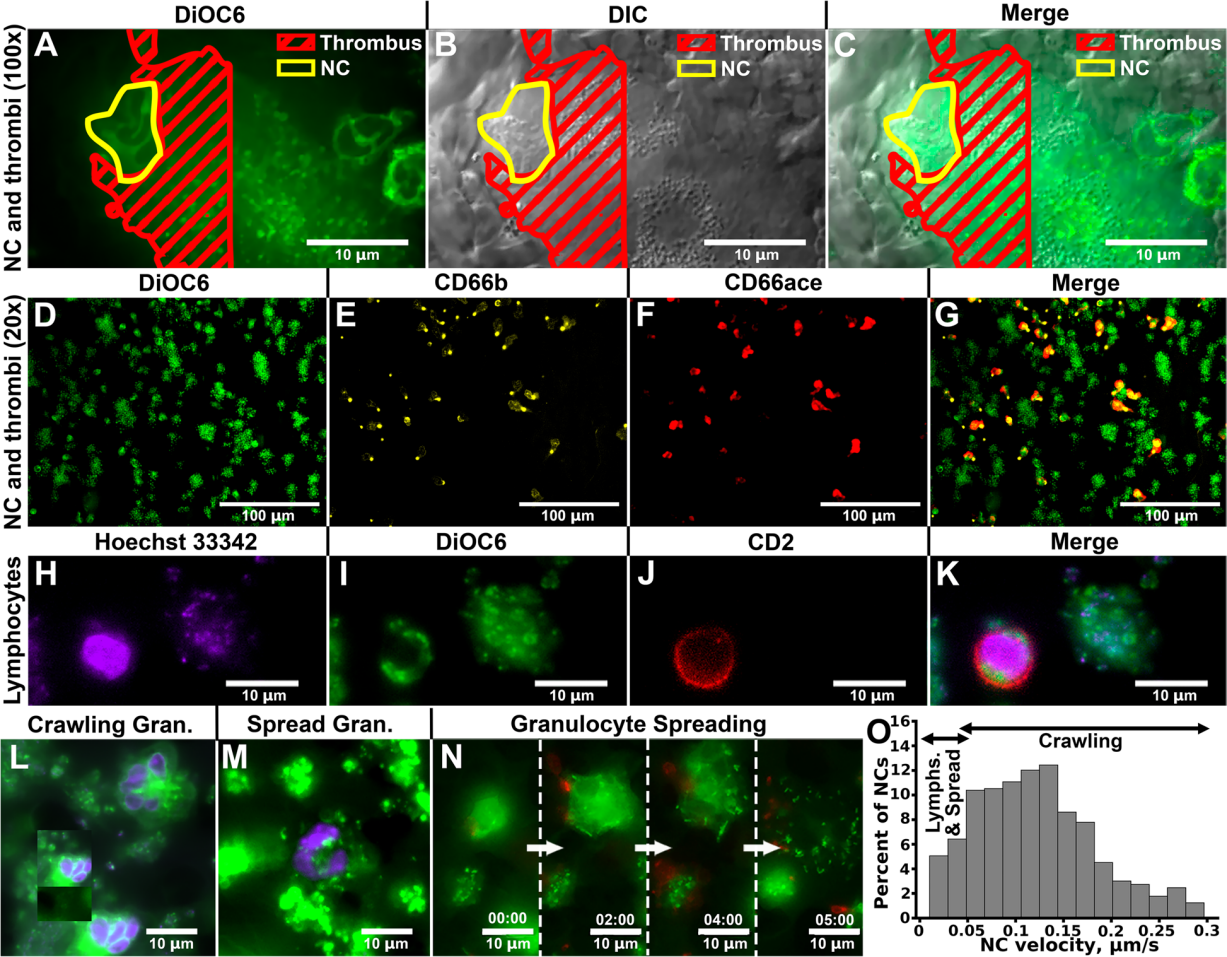
Nuclear cells (NCs) are able to crawl among the growing thrombi. **A**–**C** Thrombi (highlighted by red) with crawling NCs (highlighted by yellow) upon hirudin-anticoagulated blood perfusion through the flow chamber with fibrillar collagen fluorescent mode (DiOC6, **A**) or DIC (**B**, **C**) at × 100 magnification (raw data at 10.6084/m9.figshare.18356042.v1). **D**–**G** Thrombi with crawling NCs in the presence of DiOC6 (**D**), CD66b (**E**), and CD66ace (**F**) at × 20 magnification (**G**–merged **D**–F; raw data at 10.6084/m9.figshare.18417194.v1). **H**–**K** Among crawling CD66b and CD66ace positive cells were motionless cells with single nuclei (**H**) and clustered DiOC6 staining (**I**), which appeared to be CD2-positive (T lymphocyte marker; **J**) as well (**K**–merged **H**–**J**; raw data at 10.6084/m9.figshare.18435551.v1). **L**, **M** Different types of granulocytes were observed—crawling (**L**) and spread (**M**). **O** Granulocyte slowing down and spreading dynamics. **O** Based on the granulocyte and lymphocyte velocity distribution, it can be claimed that spread granulocyte and lymphocytes were distinctive not only by their appearance but by their velocity as well. Representative data out of *N* = 10 donors. Individual data values are given in the Additional Table 3

In order to identify the NCs, DiOC6 (Fig. 1D), the antibody to CD66ace (Fig. 1E) and the antibody against CD66ace (Fig. 1F) were used. Alternatively, blood was pre-incubated with Hoechst 33342 (H), DiOC6 (I), or anti-CD2 antibody (J). It appeared that while most of the cells were granulocytes (CD66b and CD66ace positive; Fig. 1D-G, Additional file 1: Fig. S4A-J)), a subset of cells was T lymphocytes (CD66ace, CD66b negative and CD2 positive; Fig. 1H-K, Additional file 1: Fig. S4K-O; Additional file 4: Video). Most of the crawling cells (Fig. 1L, Additional file 5: Video) appeared to have active CD11b in their posterior part (Additional file 1: Fig. S4A-E). Some of the granulocytes slowed down and spread (Fig. 1M) with fewer number of active CD11b in the middle parts of the cell (Additional file 1: Fig. S4F-J) [17]. The process of granulocyte slowing down and spreading is depicted in Fig. 1N and in Additional file 6: Video. Both granulocytes and T lymphocytes were CD18 positive; however, CD18 staining on T lymphocytes appeared to be less clustered (Additional file 1: Fig. S5). The named three cell types (crawling granulocytes, spread granulocytes and T lymphocytes) were clearly distinguishable by their DiOC6 staining pattern (Fig. 1A, I, L, M) and their velocities, namely, spread granulocytes’ and T lymphocytes’ velocity was less than 0.045 μm/s on average (Fig. 1O). The number of all types of NCs increased from 1 to 2 cells per field of view (FOV) on the 5th minute to 8 cells per FOV on the 20th minute and then did not change significantly up to 30th minute (Additional file 1: Fig. S6). Analysis of the blood contents prior and post blood perfusion revealed that nearly 50% of granulocytes participated in the process of thrombus formation in our conditions (Additional file 1: Fig. S7A-C). However, upon platelet depletion, slightly yet significantly lesser amounts of granulocytes were incorporated in the thrombus formation process in contrast to non-thrombocytopenic blood (Additional file 1: Fig. S7D-F). On the other hand, granulocyte crawling velocity appeared to be independent from the platelet count: no statistical differences were present between granulocyte crawling in normal and thrombocytopenic blood (Additional file 1: Fig. S8).

### Mediators of inflammation and platelet activators alter granulocyte behavior

**Fig. 2:**
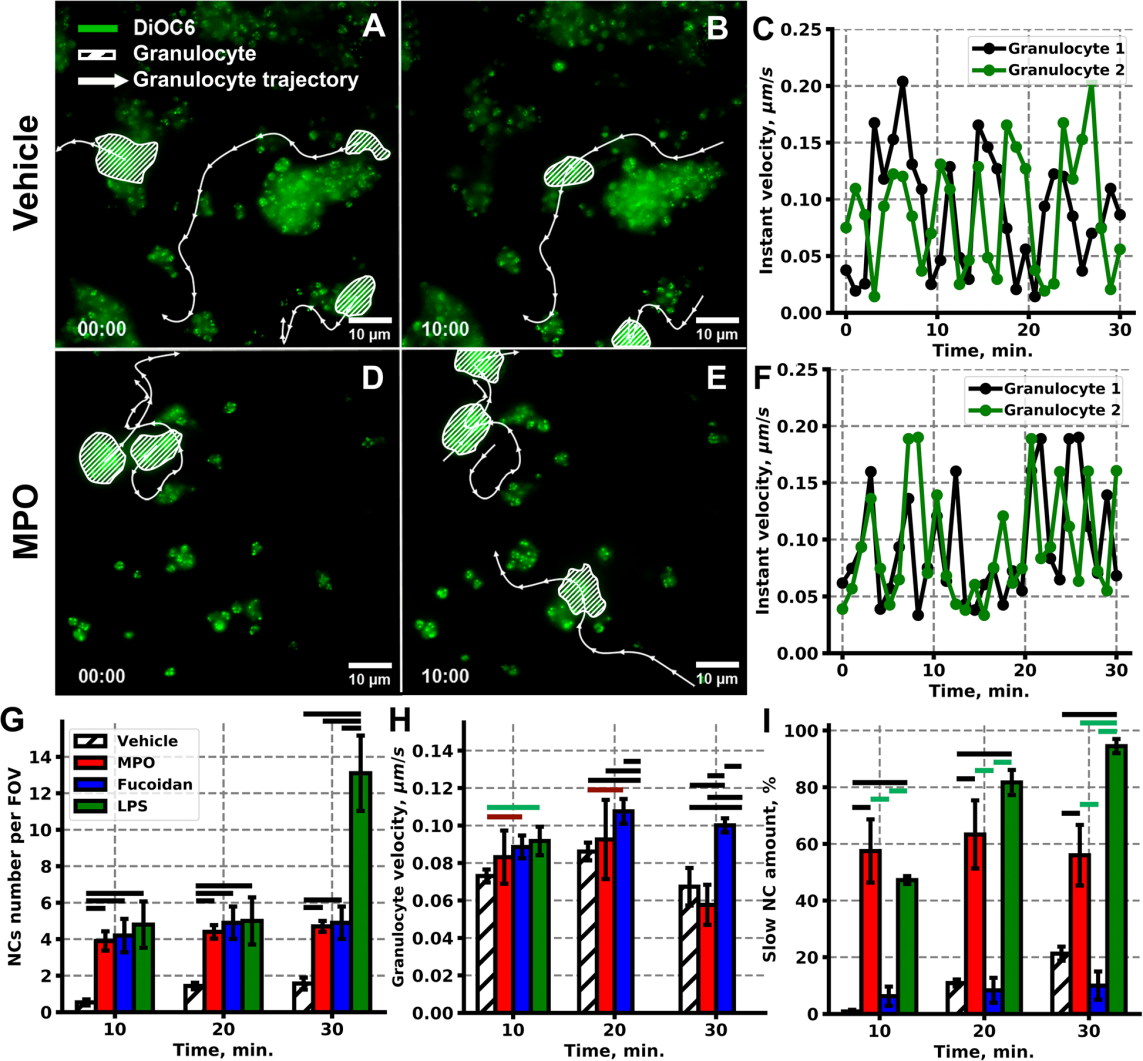
Granulocyte and platelet activators’ impact on granulocyte behavior among the growing thrombi*.***A**–**F** Granulocytes from vehicle- (**A**–**C**) or MPO- (**D**–**F**) treated blood crawl among platelet thrombi in a direct manner: **A**, **D**—initial moment; **B**, **E**—10 min after granulocyte adhesion; **C**, **F**— instant granulocyte velocity. **G**–**I** Granulocyte and platelet activators alter the number of NCs per FOV (**G**), crawling granulocyte velocity (**H**), and slow NCs amount (**I**). Statistical significance was calculated with the Mann-Whitney test; green lines correspond to *p* < 0.05, red lines correspond *p* < 0.01, black lines correspond *p* < 0.001. Statistics were calculated over 20 FOVs from *n* = 10 donors. Raw data at 10.6084/m9.figshare.18357611.v1. Individual data values are given in the Additional Table 3

In order to test whether NCs activation affected their adhesion at sites of growing thrombi, we performed experiments in the presence of leukocyte-priming agent, myeloperoxidase (MPO) [19], which is one of the key proteins, stored in granulocytes and secreted upon activation [20]. By binding to Mac-1, MPO can induce granulocyte activation in an autocrine fashion including MAPK activation, degranulation [21, 22], and adhesion [23]. It has been demonstrated previously that MPO facilitates granulocyte recruitment by its positive surface charge [24]. Furthermore, MPO-dependent granulocyte recruitment at sites of inflammation has been demonstrated both in vitro and in vivo [24]*.* MPO did not significantly affect granulocyte trajectories (Fig. 2D–F) and velocities (Fig. 2H), while significantly increasing the number of NCs per FOV (Fig. 2G) and percentage of motionless NCs (Fig. 2I) in line with the literature data on MPO impact on granulocytes [19, 22, 24]. For the combined activation of granulocytes on platelets, we used fucoidan, capable of inducing platelet degranulation (P-Selectin exposure, Additional file 1: Fig. S9) via CLEC-2 receptor [25] as well as pro-inflammatory cytokine production and apoptosis delay in granulocytes [26]. Therefore, we expect an increased attraction of granulocytes to the growing thrombi. Indeed, the number of NCs (Fig. 2G) as well as the NC crawling velocities (Fig. 2H) increased upon fucoidan treatment, while NC spreading was not significantly altered (Fig. 2I). Finally, we used lipopolysaccharides (LPS) [27] to mimic the pro-inflammatory granulocytes stimulation. LPS activate and promote NETosis via TLR4 receptor on granulocytes, while do not significantly alter platelet functioning and thrombus formation in flow chambers [28]. As expected, LPS increased granulocyte crawling velocity only at 10th minute but significantly reduced it at 20th and 30th minute (Fig. 2H), which was associated with the statistically significant increase of the numbers of slow NCs upon LPS introduction (Fig. 2I). All of the used agents altered the thrombus area; however, their effects are not uniform and should be the object of additional studies (Additional file 1: Fig. S10, S11A).

### NCs bear Annexin-V-positive platelets

**Fig. 3:**
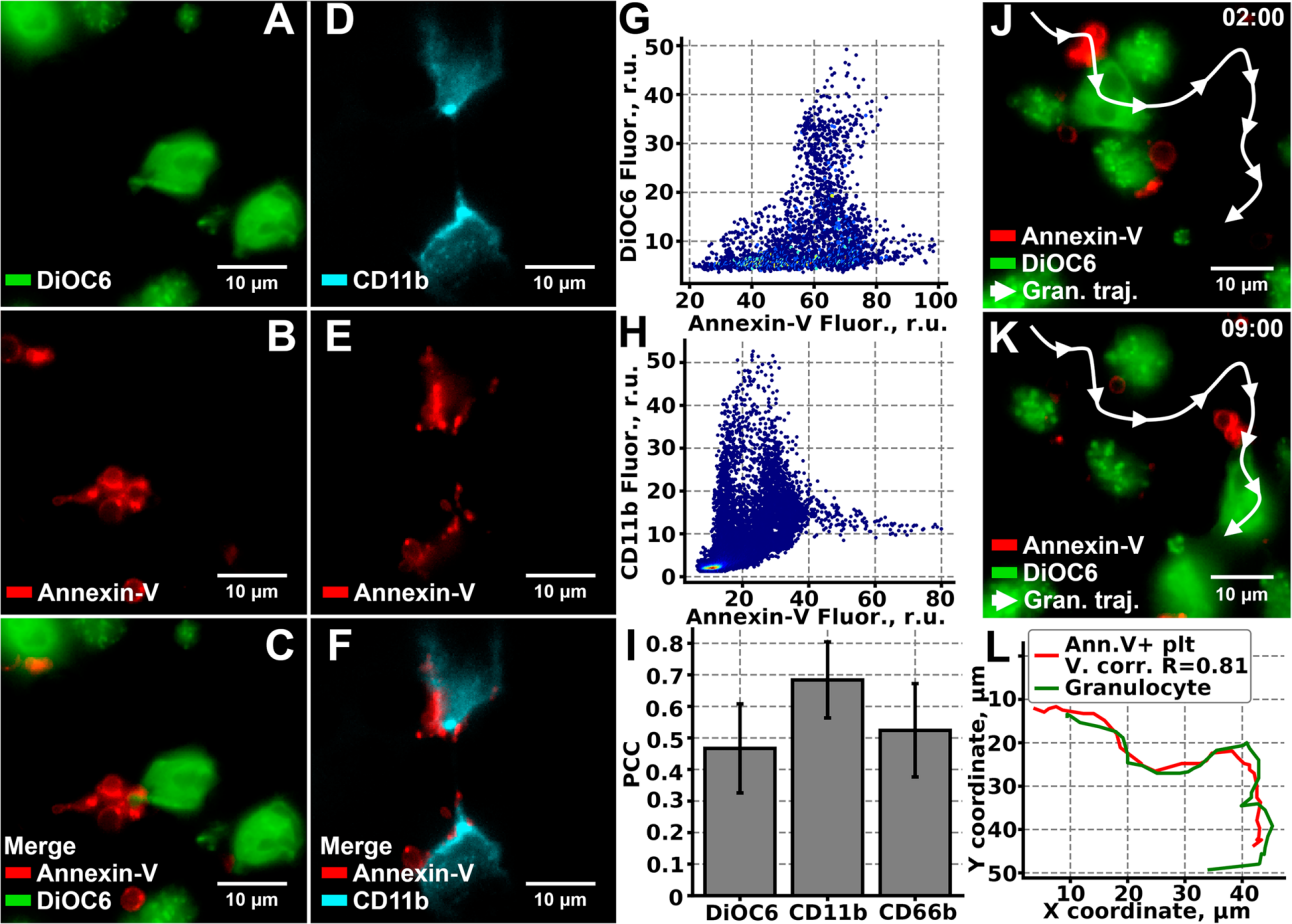
Annexin-V-positive platelets attach to the crawling granulocytes*.***A**–**C** DiOC6 (**A**) and Annexin (**B**) staining colocalized (**C**) in a manner suggesting that granulocytes bear Annexin-V-positive platelets. **D**–**F** Similar conclusions can be made based on CD11b (**D**) and Annexin-V (**E**) colocalization (**F**) analysis. **G**, **H** Scatterplots of the Annexin-V and DiOC6 (**G**) or CD11b (**H**) fluorescence intensity correlation from **C** and **F**, correspondingly (raw data at 10.6084/m9.figshare.18613442.v1). **I** Pearson’s R correlation coefficient (PCC) for Annexin-V and DiOC6, CD11b, and CD66b dyes (*n* = 50 for each pair), individual data values are given in the Additional Table 3. **J**, **K** Microscopy images of the crawling granulocyte, bearing Annexin-V-positive platelets after 2 min (**J**) and after 9 min (**K**) from the start of the observation. **L** Trajectories of the granulocyte (green curve) and the Annexin-V-positive platelet (red curve) from **J**–**K**. Typical results out on *n* = 50 trajectories

Among the key physiological functions of granulocytes in the blood flow is the removal of the phosphatidyl-serine (PS) exposing (Annexin-V-positive) cells [29]. In particular, granulocytes can form hetero-aggregates with Annexin-V-positive platelets [7], which form in the process of thrombus formation [30]. Indeed, in our experimental setting, the crawling granulocytes were associated with Annexin-V-positive particles (Fig. 3A–C). In order to identify these particles, whole blood was loaded with Hoechst 33342, anti-CD61 (specific marker of platelets) antibodies, and Annexin-V. It appeared, that the majority of granulocytes were associated with Annexin-V-positive and CD61-positive cells—procoagulant platelets (Fig. Additional file 1: 2A-E, Additional file 7: Video). Same has been observed for experiments at lower magnification (× 40): most of granulocytes appeared to be bearing Annexin-V- and CD61-positive cells (Additional file 1: Fig. S12F-I). To identify the mechanism of Annexin-V-positive platelets association with crawling NCs, we analyzed active CD11b and CD66b distribution on the crawling cells (Fig. 3 and Additional file 1: Fig. S13, correspondingly). Colocalization analysis [31] revealed that DiOC-6 (Fig. 3G) and CD66b (Additional file 1: Fig. S11D) staining did not significantly correlate (Pearson’s correlation coefficient, PCC, 0.46 ± 0.13 and 0.52 ± 0.15, correspondingly) with Annexin-V fluorescence (Fig. 3I). On the other hand, the correlation between CD11b and Annexin-V fluorescence (Fig. 3H) was significantly higher, PCC = 0.68 ± 0.12 (Fig. 3I). Therefore, platelets attach to crawling granulocytes, probably, in a CD11b-dependent manner. Furthermore, trajectories of the crawling NCs correlated to the trajectories of Annexin-V-positive platelets in most cases (Fig. 3L, Additional file 7: Video).

### Blood plasma proteins are required for granulocyte crawling among the thrombi

**Fig. 4:**
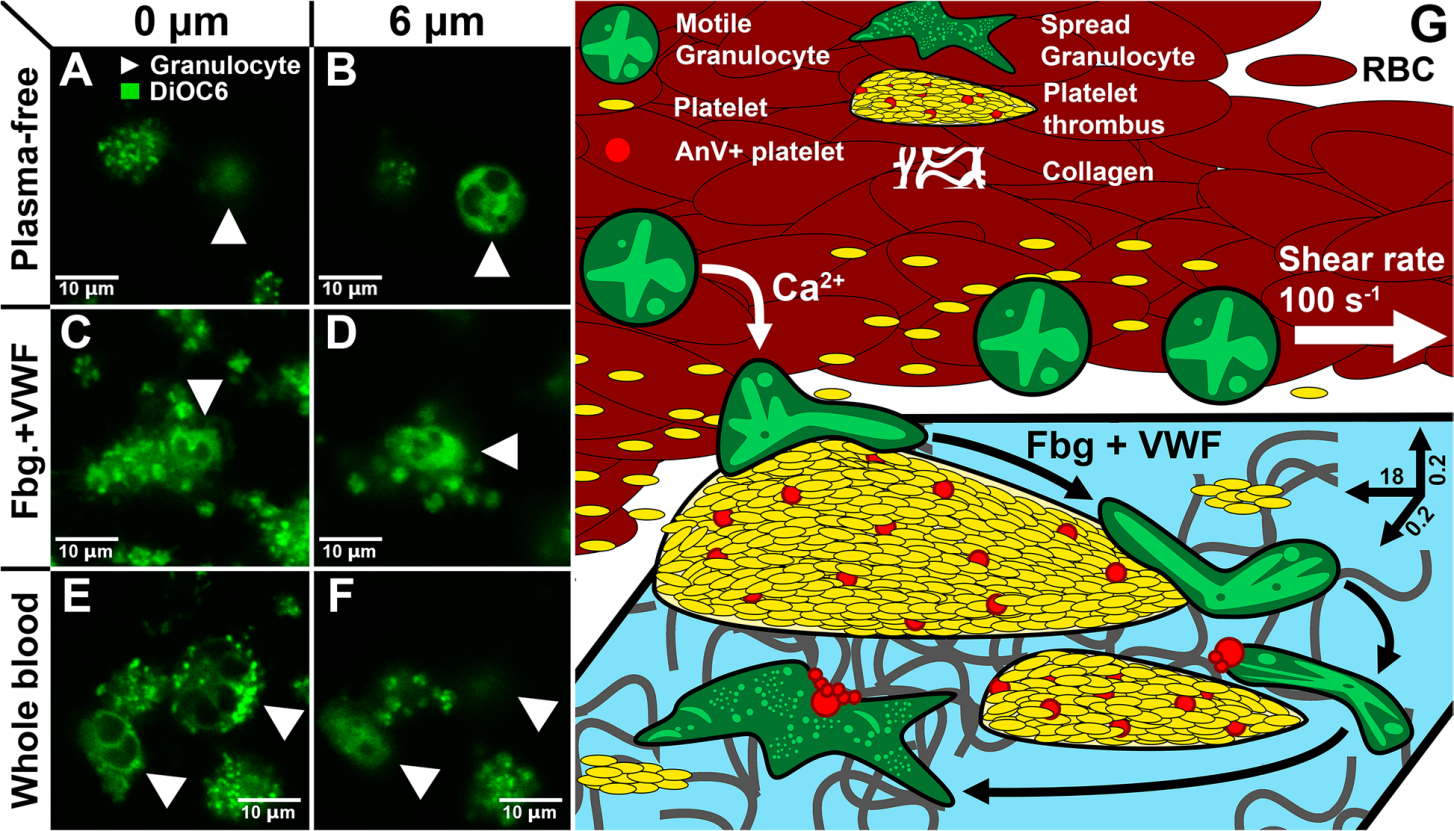
The role of blood plasma proteins in NCs incorporation into the growing thrombi*.***A**–**F** Confocal microscopy images of the growing thrombi and NCs. **A**, **B** Thrombi in the “clean” system on the collagen level (**A**) and 6 μm above (**B**). **C**, **D** Thrombi in the “clean” system with the addition of fibrinogen and VWF on the collagen level (**C**) and 6 μm above (**D**). **E**, **F** Thrombi in the whole blood on the collagen level (**E**) and 6 μm above (**F**). Representative images of *n* = 10 experiments (raw data could be found at 10.6084/m9.figshare.18358274.v1). **G** Scheme of the granulocyte behavior on the collagen covered glass coverslip (in 0.2 × 0.2 × 18 mm flow chamber): granulocytes (green cells) attach to the growing thrombi (yellow). In the presence of fibrinogen and VWF granulocytes, descend to the collagen level and collect Annexin-V-positive platelets from the thrombi in the process of crawling. Eventually, granulocytes slow down and spread on the collagen

In order to identify the mechanisms of the NC incorporation at sites of growing thrombi, we have removed plasma proteins from the whole blood by means of sequential centrifugation (see the “Methods” section). In a such “clear” system, no granulocyte crawling on the collagen surface was observed (Fig. 4A), while short-term NC attachment to the platelet covered surface and granulocyte rolling was observed 6 μm above the collagen layer (Fig. 4B, Additional file 8: Video). The introduction of 10% of physiological concentrations of fibrinogen and von Willebrand factor (VWF) resulted in the recovery of granulocyte crawling (Fig. 4C, D, Additional file 8: Video) on the collagen covered glass, yet granulocytes descended from the thrombi less readily than in the whole blood (Fig. 4E, F, Additional file 8: Video). Based on these findings, we propose the following scheme of the events: NCs from the blood flow attach to the growing thrombi in the presence of calcium and descend onto the collagen level via fibrinogen and VWF, where granulocytes crawl, bearing Annexin-V-positive platelets and eventually slow down and spread (Fig. 4G).

### Crawling of WAS patients’ granulocytes is altered in comparison to healthy donors

Altogether, the observed features of granulocytes crawling are consistent with the granulocyte chemotaxis phenomenon. Therefore, we assumed it should be altered in cells with defective cytoskeleton. Wiskott-Aldrich syndrome is a genetic disease, caused by WAS gene mutations and alteration of cytoskeleton of both immune cells and platelets [32]. WAS is mainly characterized by immunodeficiency, microthrombocytopenia, and autoimmune/oncological predisposition [14].

**Fig. 5:**
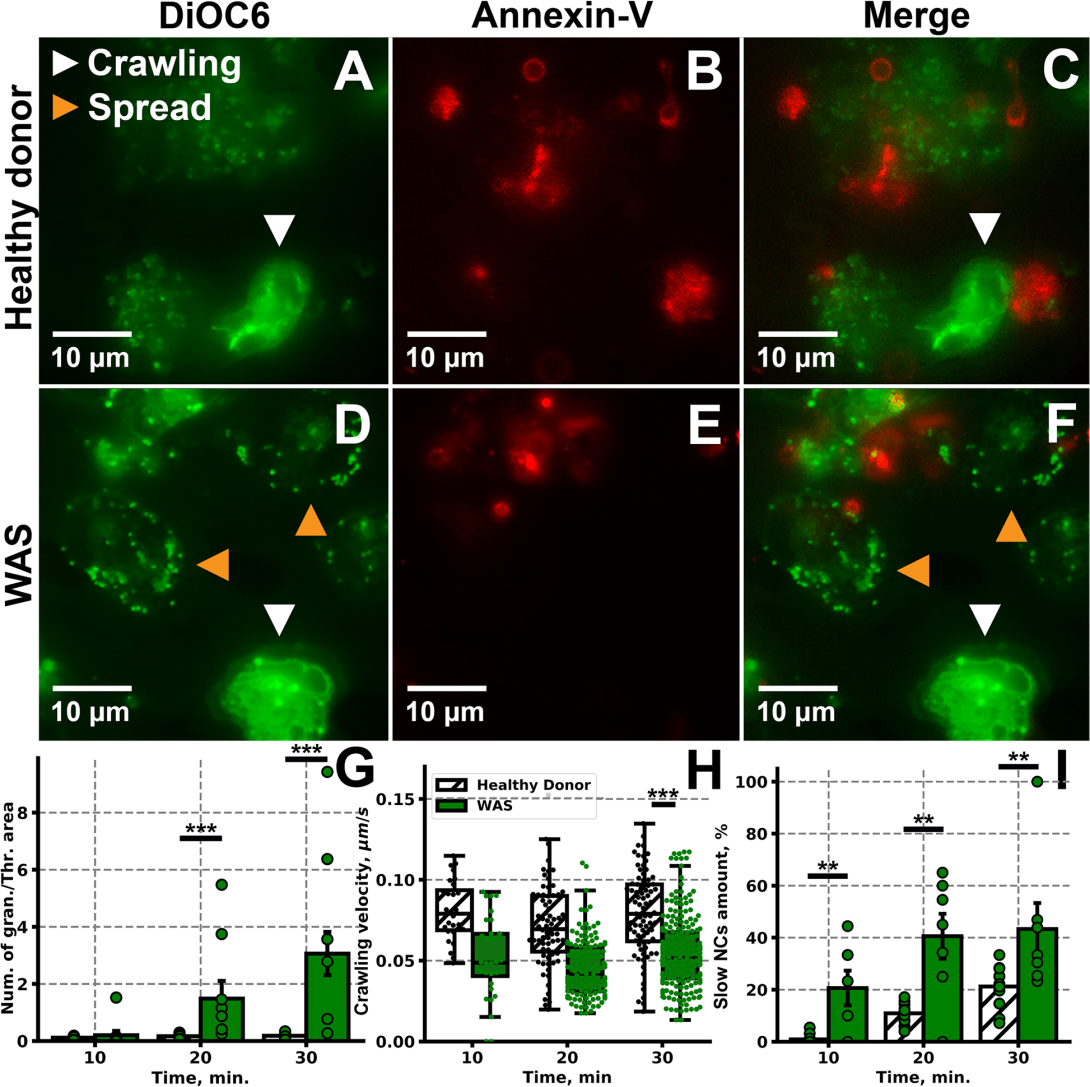
Comparison of healthy donors’ and WAS patients’ NCs behavior. **A**–**C** Typical for healthy donors FOV with growing thrombi and crawling granulocytes. **D**–**F** Typical for healthy donors FOV with growing thrombi and crawling granulocytes. **G**–**I** Quantitative comparison of the granulocyte behavior of healthy donors (white) and WAS patients (green): the ratio of granulocyte number to thrombus area (**G**), average velocities of the crawling granulocytes (**H**), amounts of spread granulocytes and lymphocytes (slow NCs) (**I**) for *n* = 10 healthy donors and *n* = 7 WAS patients (raw data could be found at 10.6084/m9.figshare.18359408.v1, individual data values are given in the Additional Table 3). Statistical significance was calculated with Mann-Whitney test, ** corresponds to *p* < 0.01, *** corresponds to *p* < 0.001

Typical FOVs of healthy donors and WAS patients are shown in Fig. 5A–C and D–F, correspondingly. The number of granulocytes per FOV was increased (Additional file 1: Fig. S11C) in WAS patients in comparison to healthy donors. This resulted in a significantly increased ratio of granulocyte number to thrombus area in WAS samples (Fig. 5G, Additional file 1: Fig. S11B). On the other hand, crawling granulocytes of WAS patients were significantly slower than crawling granulocytes of healthy donors (Fig. 5G). Finally, slow NC amount was significantly increased in WAS samples in comparison to healthy donor samples (Fig. 5H). Thus, it can be claimed WAS granulocytes and MNCs are more prone to the collagen adhesion and spreading. In order to check, whether granulocyte velocity are not altered due to thrombocytopenia or neutropenia, we partially supplemented leukocyte rich plasma with Tyrode’s, analogous to our earlier study [33]. It appeared, that in the “neutropenic” conditions no alterations in the granulocyte crawling were present (Additional file 1: Fig. S11D).

Finally, experiments with “hybrid” samples were performed. Blood samples from patients with WAS were platelet-depleted and substituted with healthy donor’s platelets. Washed healthy donor platelets were loaded with Fura-Red in order to distinguish them from the native WAS patient cells (Additional file 1: Fig. S14 A-O). It appeared that the observed thrombi were Fura-Red positive, which allowed us to claim that platelet replacement was effective. Alternatively, effectiveness of the replacement was controlled using a cell counter: platelet count in the whole blood dropped upon depletion and then increased upon healthy donor platelet addition (Additional file 1: Fig. S14P). Furthermore, platelet size also increased upon replacement, what additionally confirms validity of the developed experimental setting, as WAS patients typically have smaller platelets (Additional file 1: Fig. S14R). Granulocyte numbers in patients remained intact (Additional file 1: Fig. S13S). Average velocities of the granulocytes of WAS patients in the presence of WAS platelets and healthy donor platelets did not differ significantly (Additional file 1: Fig. S14T), while the amount of spread granulocytes and lymphocytes even increased (Additional file 1: Fig. S14U). As granulocytes of the WAS patients in were generally slower than granulocytes of healthy donors (Fig. 5G), it can be stated that substitution of the defective platelets of the patients by healthy donor platelets does not alter WAS patient granulocyte crawling.

## Discussion

Here, we developed an ex vivo approach to study granulocyte involvement into the thrombus formation. We observed leukocytes rolling, crawling, and arrest in thrombus formation (Fig. 1). The granulocytes behavior was affected by MPO, fucoidan, and LPS (Fig. 2). Most of granulocytes were bearing procoagulant platelets, presumably, in CD11b-dependent manner (Fig. 3). The observed granulocyte incorporation in growing thrombi appeared to be mediated by plasma proteins—fibrinogen and VWF (Fig. 4). For WAS patients, lower crawling velocities and higher relative number of granulocytes were observed (Fig. 5). Thus, the proposed ex vivo experimental setting allows to observe granulocytes activity in near-physiological conditions.

Here, we provide evidence that granulocyte behavior during thrombus formation could be studied ex vivo in parallel-plate flow chambers at wall shear rates less than 200 s^−1^ and in presence of physiological calcium level (Fig. 1, Additional file 1: Fig. S1, Table 1) as well as a substantial amount of fibrinogen and VWF (Fig. 4). We propose hirudin as the most convenient anticoagulant for such studies, because citrate recalcification significantly affects reproducibility of the experiments [34]. Heparin could be an attractive option; however, heparin impact on the immune cell behavior is well established [19, 22, 35, 36] and was confirmed in this study as well (Additional file 1: Fig. S3, Table 1).

The first quantitative characteristic of granulocytes proposed here is their movement velocity, which appears to be 0.1 ± 0.02 μm/s on the average, with instant velocities reaching 0.5 μm/s (Table 1). These values increased upon priming of granulocytes with various agents (Fig. 2) and decreased in WAS samples (Fig. 5). Previously, C. Jones et al. showed that human neutrophils migrated towards LTB4 with an average velocity of 0.39 ± 0.09 μm/s [37]. While Jones et al. used a constant gradient of chemoattractant, in our experimental setting, there were several target areas of attraction for granulocytes, which might explain non-monotonous movements of granulocytes in the current study (Fig. 2, Additional files 2 and 3: Video). In another study, M. Weckmann et al. observed the velocity of neutrophil migration on fibronectin in the presence of IL-8, fMLP, and LTB4 [38], where leukocyte velocity appeared to be 0.11 ± 0.12 μm/s, the same as in our study in the presence of the priming reagents (Fig. 2).

The second quantitative characteristic observed here is the percentage of spread granulocytes and attached T lymphocytes. It has been observed that with the flow of time, granulocytes slow down and spread (Fig. 1, Additional file 6: Video); however, it cannot be claimed that these cells are undergoing NETosis, as it has been demonstrated that NETosis usually occurs later than 30 min after activation [39]. Analysis of the active CD11b distribution revealed that while active CD11b were present at posterior parts of the crawling cells (Additional file 1: Fig. S4B, Additional file 5: Video), in the spread cells, CD11b was less active and located in the middle part of the cells. This was in agreement with previously published data [40]. It is noteworthy that not only granulocytes participated in the thrombus formation in our setting: a plethora of T lymphocytes were present as well (Additional file 1: Fig. S4, S5), and thus, their role in thromboinflammation is to be respected as well. Detailed study of the T lymphocyte activity in the given setting should be the object of further studies.

The third semi-quantitative characteristic is the total number of granulocytes per field of view (Fig. 2). This value depends on the platelet functioning, because it is well-known that platelets activate and attract leukocytes by platelet-leukocyte interactions during thromboinflammation [1, 9, 41].

Hereby, we assume that our approach can be used for the assessment of platelet-granulocyte interplay in different conditions, including hematological and immunological disorders, such as WAS. We observed impaired granulocyte activity in WAS patients (Fig. 5), consistent with the reduced integrin-dependent degranulation and respiratory burst [42]. The enhanced granulocyte recruitment to the growing thrombi (Fig. 5G) can be the result of the higher proportion of procoagulant platelets in WAS patients [43, 44]. Supplementation of the WAS-patient platelets by healthy donor platelets did not result in a significant increase in the granulocyte velocity that allows to claim that granulocyte motility in WAS patients is caused by WASP deficiency instead of platelet dysfunction (Additional file 1: Fig. S14). It should be noted that additional animal studies could be helpful in order to determine whether direct WASP mutations affect NC crawling in the absence of WASP mutations in platelets.

Several other new findings of platelet-granulocyte interplay were observed in our study. First, we observed procoagulant platelets attached to the moving granulocytes (Fig. 3, Additional file 1: S12). Procoagulant platelets are hyperactivated platelets that underwent mitochondria-dependent necrosis and exposed phosphatidylserine on their surface in the first minutes upon activation [45]. These cells have compromised cytoskeleton what results in their increase in size and loss of most of adhesive integrins [43]. Procoagulant platelets in our conditions attracted granulocytes in the manner common for any tissue debris (Fig. 3), as demonstrated in several studies [46].

Based on our results, we also claim that the mechanism of the granulocyte involvement in thrombus formation is dependent on plasma proteins (fibrinogen and VWF) (Fig. 4A–F). This is in line with findings of Constantinescu-Bercu A. et al. [47] who demonstrated VWF- and platelet integrin αIIbβ3-dependent activation of neutrophils and with findings of Ghasemzadeh M. et al. [48], who confirm the essential role of fibrin in intravascular leukocyte trafficking. Additionally, the VWF role in the recruitment of leukocytes during thromboinflammation has been previously established [49].

## Conclusions

In this study, we report the phenomenon of the granulocyte crawling among the growing thrombi ex vivo. We claim that granulocyte characterization can be used for a more in-depth analysis of the mechanisms of immunological and hematologic diseases. Based on our own experimental assays, we propose a scheme of granulocyte participation in thrombus formation: (1) granulocytes attach to growing thrombi in a calcium-dependent manner, (2) granulocyte descent to collagen level is mediated by fibrinogen and VWF, (3) descended granulocytes collect annexin-V-positive platelets from the growing thrombi, and (4) granulocytes eventually slow down and arrest (Fig. 4).

## Methods

### Aim

The key aim of the study was to establish the experimental setting for the ex vivo observation and characterization of the immune cell participation in the process of venule-thrombus formation by means of fluorescent microscopy.

### Materials

The sources of the materials were as follows: Annexin V-Alexa Fluor 647 (BioLegend, San Diego, CA), DiOC-6, Fucoidan from *Fucus vesiculosis*, HEPES, bovine serum albumin, lipopolysaccharides from *E. coli* O111:B4, human fibrinogen, Hoechst-33342 (Sigma-Aldrich, St Louis, MO); CD11b-FITC, CD18-APC, CD66ace-Alexa647, CD66b-PE, CD2-APC (Sony Biotechnology, San Jose, CA), fibrillar collagen type I (Chrono-Log Corporation; Havertown; USA); human von Willebrand factor (VWF) was a kind gift of Prof. Pierre Mangin (NSERM, Etablissement Français du Sang-Grand Est, UMR_S1255, Fédération de Médecine Translationnelle de Strasbourg, Université de Strasbourg, France). The HL-60 cell line (promyelocytic leukemia) was used as a source of myeloperoxidase (MPO). MPO was isolated, as described in [50]. LAL-test was performed on human fibrinogen and VWF using LAL-kit Lonza (QCL-1000) in order to ensure absence of the endotoxin contamination (Additional file 1: Tables S1, S2).

### Blood collection and handling

Blood collection was performed under the protocol approved by the free CTP PCP RAS Ethical Committee (protocol #1 from 12.01.2018), and written informed consents were obtained from all donors and patients. Blood was collected from healthy adult volunteers (*n* = 35, men and women 18–35 years old) into Vacuette© sodium citrate (3.8 % v/v) or lithium heparin (18 I.U./ml blood) or Sarstedt-Monovette© hirudin (525 ATU/ml blood) vacuum tubes. Experiments were performed within 3 h after blood collection. For the assays involving Wiskott-Aldrich syndrome patients, blood was collected from healthy pediatric donors (*n* = 12) or from patients with Wiskott-Aldrich syndrome (*n* = 10) into Sarstedt-Monovette hirudin (525 ATU/ml blood) tubes.

For the experiments with “hybrid” and “plasma-free” settings, blood samples were purified from plasma proteins by 3 sequential centrifugations of citrated whole blood for 10 min by 1000 *g* with supplementation of the plasma by Tyrode’s calcium-free buffer. Final supplementation was performed by Tyrode’s calcium buffer. For artificial thrombocytopenia studies, whole citrated blood of healthy donors was centrifuged at 100 *g* for 8 min. PRP was collected above the buffy coat and centrifuged at 1000 for 10 min. The resultant supernatant was then collected and returned to the whole blood. For control experiments, PRP was returned to the blood without additional centrifugation. Platelet count was monitored using Drew D3 cell counter (Drew Scientific, USA). For WAS-hybrid patient studies patients’ samples were depleted from platelets in the same manner. Platelets of healthy donors were washed by sequential centrifugations as described earlier [45] (100 *g* 8 min for PRP and then two centrifugations for 10 min at 1000 *g* with resuspension in Tyrode’s buffer) and concentrated 10 times above initial. Healthy donor platelets were then added to the WAS patient platelet-depleted blood. Prior to the experiments, the sample was recalcified to achieve free calcium concentration of 2.5 mM.

### Fluorescent microscopy

Parallel-plate flow chambers were described previously [30]. Channel parameters were as follows: 0.2 × 18 × 0.206 mm. Glass coverslips were coated with fibrillar collagen type I (0.2 mg/ml) for 1 h 30 min at 37 °С, washed with distilled water and then inserted into the flow chambers. Blood was perfused through the parallel-plate chambers over collagen-coated (0.2 mg/ml) surface with wall shear rates 100 s^−1^ [51]. Thrombus growth and leukocyte crawling were observed in DIC/epifluorescence modes with an inverted Nikon Eclipse Ti-E microscope (100x/1.49 NA TIRF oil objective).

### Data analysis

Nikon NIS-Elements software was used for microscope image acquisition; ImageJ (http://imagej.net/ImageJ) was used for image processing. ImageJ manual tracking plugin was used for manual granulocyte tracking, and the Coloc2 plugin for fluorescence colocalization analysis plugin was utilized. For automated cell tracking, particle tracking algorithm described in [52] was utilized. The algorithm was based on Python trackpy v 0.4.2 library. First, particle tracking was performed, and then the tracks belonging to leukocytes were selected manually. The platelet thrombus area was calculated as the percentage of the screen covered by platelet thrombi upon the subtraction of the area of crawling cells. Tracking Code listing and program operation examples can be found in the data availability statement below.

### Statistics

All experiments were performed at least in triplicate with platelets from different donors. Statistical analysis was performed using Python 3.6; all statistical details are provided in the figure legends.

## Supplementary Information

**Additional file 1: Figure S1.** Comparison of the anti-coagulant impact on NC incorporation to the growing thrombi. **Figure S2.** Instant NC velocities and averaged velocities. **Figure S3.** Comparison between heparin and hirudin impact on NCs. **Figure S4.** Classification of the NC cells among the thrombi. **Figure S5.** Analysis of the CD11b/CD18 distribution in granulocytes and MNCs. **Figure S6.** The number of NCs per FOV increased gradually up to the 20th minute of the experiment. **Figure S7.** Blood cell composition changes after perfusion through flow chamber with fibrillar collagen or BSA. **Figure S8.** Impact of platelet depletion on granulocyte crawling velocity. **Figure S9.** Fucoidan impact on platelet P-selectin exposure in growing thrombi. **Figure S10.** Granulocyte movement around thrombi. **Figure S11.** Thrombus area and percentage of highly activated granulocytes in the presence of leukocyte activators and in patients with WAS. **Figure S12.** Crawling granulocytes bear Annexin-V positive platelets. **Figure S13.** Annexin-V and CD66b staining. **Figure S14.** Assay of the granulocyte crawling in the blood of WAS patients in the presence of healthy donor platelets. **Table S1.** LAL-test results for endotoxin characterization in the protein samples. **Table S2.** Calibration of the LAL-test system.

**Additional file 2.** Video: Blood anticoagulant impact on NC behavior and thrombus growth. Comparison of the typical Hoechst 33342 and DiOC6 stained NC behavior alongside thrombus growth in the EDTA, sodium citrate, sodium citrate + calcium, heparin, and hirudin anticoagulation.

**Additional file 3.** Video: Types of NC motion. Typical rolling, crawling, and slow NCs were stained with Hoechst 33342 (magenta) and DiOC6 (green).

**Additional file 4.** Video: Lymphocyte identification. Videos show that slow NCs with single nuclei (stained by Hoechst 33342 – magenta and DiOC6 – green) are CD2 positive (red) and are thus T-lymphocytes.

**Additional file 5.** Video: Granulocyte crawling. Crawling cells with polymorphonuclear structure (Hoechst 33342 – magenta) are CD11b (blue) and CD66ace (red) positive. Thus, in the given setting, the observed crawling cells are granulocytes.

**Additional file 6.** Video: Changes in DiOC6 distribution upon granulocyte activation. Videos of the granulocyte (DiOC6 stained – green) crawling, slowing down and subsequent spreading among the growing thrombi.

**Additional file 7.** Video: Granulocytes bear Annexin-V+ platelets. Videos of Hoechst 33342 (magenta) labeled granulocytes, which tear away from platelet thrombi (CD61 – blue) procoagulant Annexin-V (red) positive platelets.

**Additional file 8.** Video: Plasma protein impact on NC attraction to thrombi. Typical timelapse and Z-stack videos of granulocyte behavior upon blood plasma depletion in the absence or the presence of VWF and fibrinogen, accompanied by the standard whole blood experiments.

**Additional file 9: Additional Table 3.** Individual data values for Figs. 1, 2, 3, 5, S3, S6, S7, S8, S9, S11, S14.

## Data Availability

All data generated or analyzed during this study are included in this published article, its supplementary information files, and publicly available repositories. Python code for the calculation of the crawling granulocyte velocity is uploaded to GitHub repository (minimal: python 3.4; platform independent; requirements – trackpy and anaconda packages for python 3.4 and above): https://github.com/CTPSignalingLab/granulocyte_crawling.git. Raw microscopy data could be found at https://figshare.com/projects/Granulocyte_involvement_in_thrombus_formation/130217.

## Abbreviations

MPO: Myeloperoxidase
LPS: Lipopolysaccharides from *E. coli* O111:B4
VWF: von Willebrand factor
PS: Phosphatidylserine
GP: Glycoprotein
CD: Cluster of differentiation
FOV: Field of view
WAS: Wiskott-Aldrich syndrome
NCs: Nuclear cells
