# Supplementary material for: Ex vivo observation of granulocyte activity during thrombus formation
Source: BMC Biol. 2022 Feb 7;20:32. doi: 10.1186/s12915-022-01238-x (PMC8819951; doi:10.1186/s12915-022-01238-x)
Supplement: Supplementary file 1 — Additional file 1: Figure S1. Comparison of the anti-coagulant impact on NC incorporation to the growing thrombi. Figure S2. Instant NC velocities and averaged velocities. Figure S3. Comparison between heparin and hirudin impact on NCs. Figure S4. Classification of the NC cells among the thrombi. Figure S5. Analysis of the CD11b/CD18 distribution in granulocytes and MNCs. Figure S6. The number of NCs per FOV increased gradually up to the 20th minute of the experiment. Figure S7. Blood cell composition changes after perfusion through flow chamber with fibrillar collagen or BSA. Figure S8. Impact of platelet depletion on granulocyte crawling velocity. Figure S9. Fucoidan impact on platelet P-selectin exposure in growing thrombi. Figure S10. Granulocyte movement around thrombi. Figure S11. Thrombus area and percentage of highly activated granulocytes in the presence of leukocyte activators and in patients with WAS. Figure S12. Crawling granulocytes bear Annexin-V positive platelets. Figure S13. Annexin-V and CD66b staining. Figure S14. Assay of the granulocyte crawling in the blood of WAS patients in the presence of healthy donor platelets. Table S1. LAL-test results for endotoxin characterization in the protein samples. Table S2. Calibration of the LAL-test system. [file 12915_2022_1238_MOESM1_ESM.pdf]

Additional Figures and Tables

Title: Ex vivo observation of granulocyte activity during thrombus formation

Authors: Daria S. Morozova[1]†, Alexey A. Martyanov[2-5]†, Sergei I. Obydennyi[2-4], Julia-Jessica D. Korobkin[2], Alexey V. Sokolov[6], Ekaterina V. Shamova[7], Irina V. Gorudko[7], Anna L. Khoreva[4], Anna Shcherbina[4], Mikhail A. Panteleev[2-4], Anastasia N. Sveshnikova[2-4,8#]

1- Faculty of Basic Medicine, Lomonosov Moscow State University, 27/1 Lomonosovsky av., Moscow, Russia, 119991

2- Faculty of Physics, Lomonosov Moscow State University, 1/2 Leninskie gory, Moscow, Russia, 119991

3- Center for Theoretical Problems of Physicochemical Pharmacology, Russian Academy of Sciences, 30, Srednyaya Kalitnikovskaya str., Moscow, Russia, 109029

4- National Medical Research Centre of Pediatric Hematology, Oncology and Immunology named after Dmitry Rogachev, 1 Samory Mashela St, Moscow, Russia, 117198

5- Institute for Biochemical Physics (IBCP), Russian Academy of Sciences (RAS), Russian Federation, 4, Kosyigina str., Moscow, Russia, 119334

6- Institute for Experimental Medicine, 12 Acad. Pavlova str., Saint Petersburg, Russia, 197376

7 - Department of Biophysics, Faculty of Physics, Belarusian State University, Minsk

8 - Department of Normal Physiology, Sechenov First Moscow State Medical University, 8/2 Trubetskaya St., Moscow, Russia, 119991

# corresponding author: a.sveshnikova@physics.msu.ru

† these authors contributed equally

Additional Tables

Table S1. LAL-test results for endotoxin characterization in the protein samples.

	A405 Run 1	A405 Run 2	A405 Run 3	Mean	Endotoxin, EU/ml
**VWF**	0.061	0.062	0.06	0.0601	-0.00408
**Fibrinogen**	0.062	0.058	0.06	0.06	-0.0049

Table S2. Calibration of the LAL-test system

Endotoxin, EU/ml	A405 Run 1	A405 Run 2	A405 Run 3	Mean
**0**	0.061	0.06	0.062	0.061
**0.025**	0.082	0.081	0.084	0.0823
**0.1**	0.159	0.164	0.155	0.159
**0.25**	0.409	0.41	0.421	0.411
**0.5**	0.709	0.728	0.712	0.716
**1**	1.246	1.259	1.295	1.267

Additional Figures

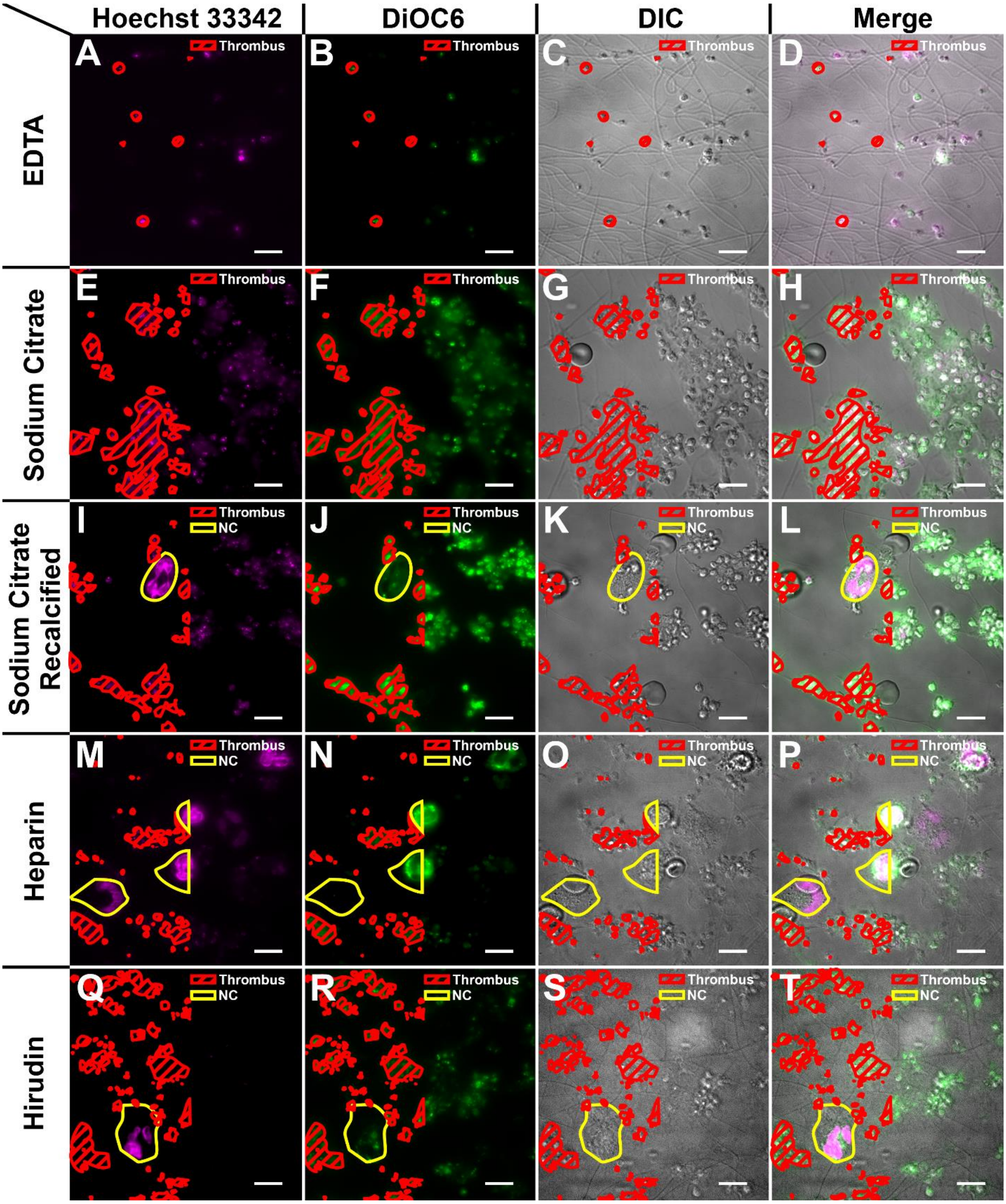

Figure S1. Comparison of the anti-coagulant impact on NC incorporation to the growing thrombi. Whole blood anti-coagulated with EDTA (A-C), Sodium citrate (E-H), Sodium citrate with recalcification (I-L), heparin (M-P) and hirudin (Q-T). Thrombi are highlighted in red, NCs are highlighted in yellow. Scalebars represent 10 µm. Typical results out of N=5 healthy donors (https://doi.org/10.6084/m9.figshare.18356042.v1).

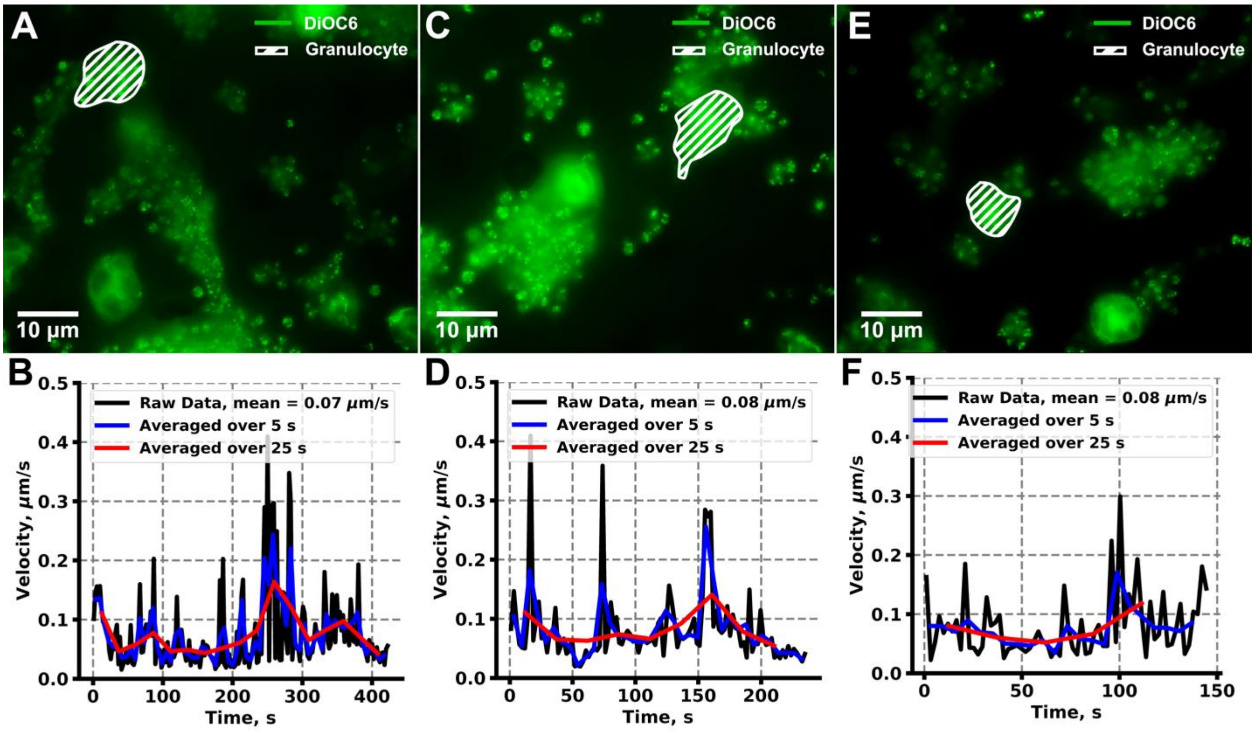

Figure S2. Instant NC velocities and averaged velocities. Epifluorescence (DiOC6 stain – green) mode was used. Images and corresponding velocity of healthy different granulocytes of a healthy donor.

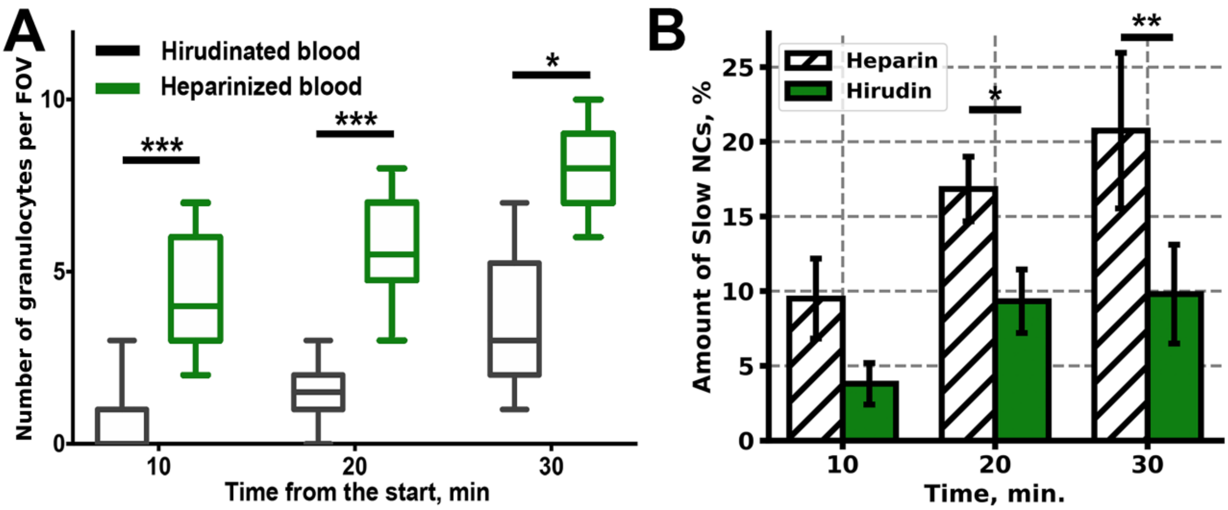

Figure S3. Comparison between heparin and hirudin impact on NCs. (A) –comparison of NC number in heparinazed and hirudinated blood; (B) – percentage of highly spread granulocytes in whole blood with different anticoagulants at different time intervals. Statistical significance was calculated with Mann-Wittney test, asterisk (*) indicates p = 0.01. Individual data values in the Additional Table 3.

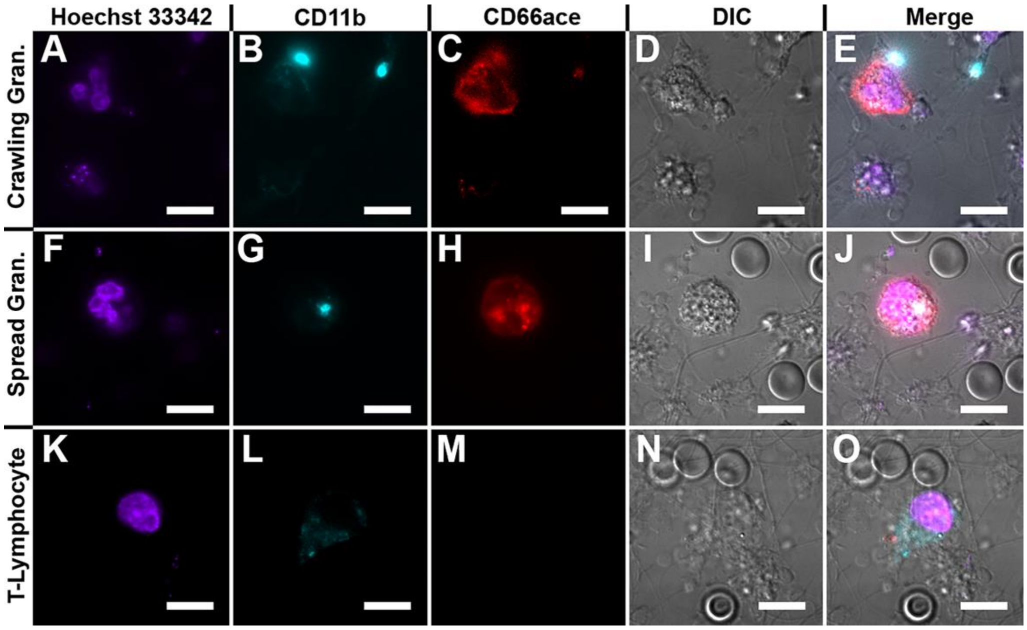

Figure S4. Classification of the NC cells among the thrombi. (A-E) Typical crawling granulocyte has a polymorphonuclear structure (A), posterior distribution of active CD11b (B) and is CD66ace positive (C). (F-J) Slowed down and spread granulocyte has polymorphonuclear structure (F), has lesser amounts of active CD11b located in the middle part of the cell (G) and is CD66ace positive. (K-O) Adherent T-lymphocytes have distinctive single nuclei (K), the least amount of active CD11b distributed more evenly than in granulocytes (L) and has no CD66ace staining (M). Scalebars represent 10 µm. Typical results out of N=5 healthy donors (https://doi.org/10.6084/m9.figshare.18360128.v1).

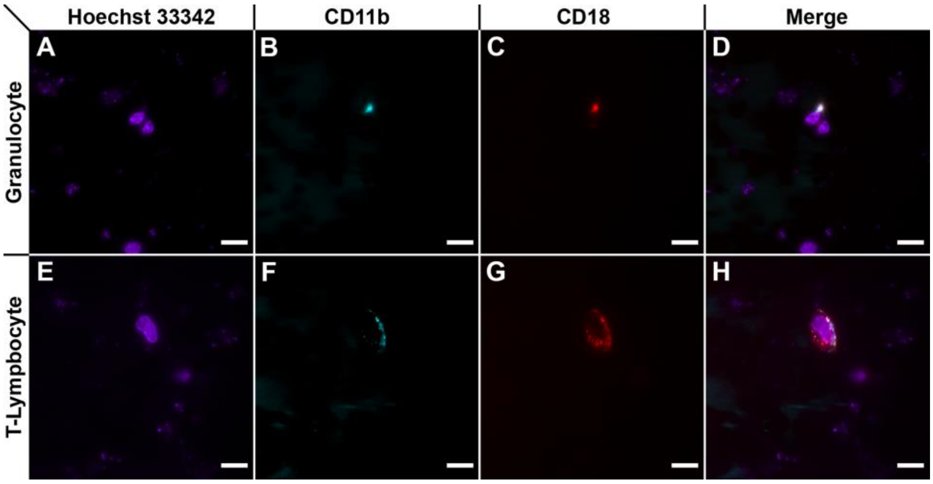

Figure S5. Analysis of the CD11b/CD18 distribution in granulocytes and MNCs. (A-D) Granulocytes had polymorphonuclear structure (A), clustered active CD11b (B) and CD18 (C). (E-H) MNCs had a single distinctive core (E), more evenly distributed active CD11b (F) and CD18 (G). Scalebars represent 10 µm. Typical results out of N=5 healthy donors (https://doi.org/10.6084/m9.figshare.18417980.v1).

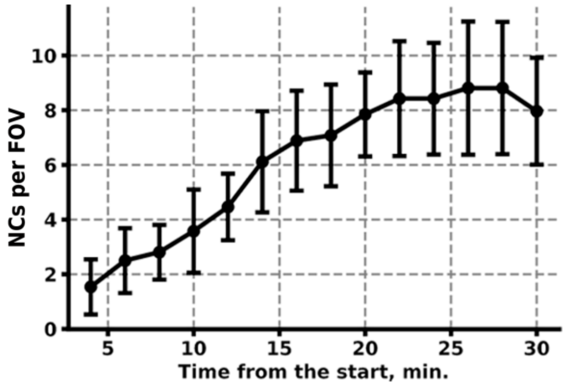

Figure S6. The number of NCs per FOV increased gradually up to the 20th minute of the experiment. Representative data out of N = 10 donors. Individual data values in the Additional Table 3.

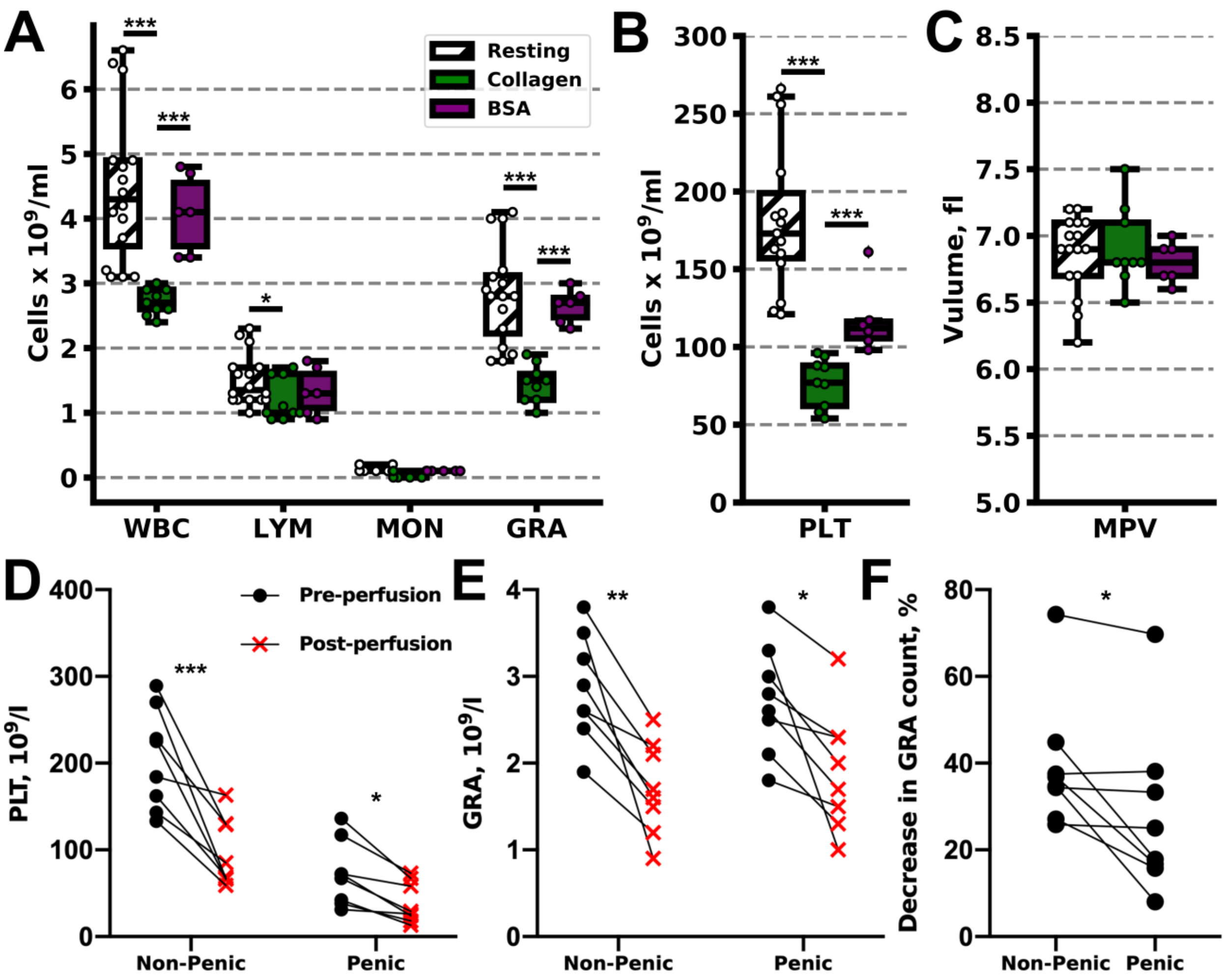

Figure S7. Blood cell composition changes after perfusion through flow chamber with fibrillar collagen (green boxes) or BSA (violet boxes). A – upon perfusion on collagen number of granulocytes decreased 46%, while no significant differences were noted upon perfusion through BSA covered glass. B – decrease in platelet amount was significant in both cases, however, perfusion over collagen resulted in a more pronounced decrease of platelet count. C – Platelet volume was not altered upon perfusion. N=12 healthy donors were included in the resting group, N=8 donors in collagen and N=6 in BSA. Statistical significance was calculated based on Mann-Whitney criteria. * - $p < 0.05$; ** - $p < 0.01$; *** - $p < 0.001$. D-F. Impact of the platelet depletion from the whole blood on the granulocyte count upon perfusion through the collagen covered coverslips. Platelet (D) and granulocyte (E) counts in non-depleted (Non-

Penic) and depleted (Penic) blood both times decreased significantly upon perfusion through the flow chamber. However, decrease in the granulocyte count upon perfusion was weakly, yet significantly less pronounced in the thrombocytopenic blood (F). N=8 donors. Statistical significance was calculated based on paired t-test criteria. * - p < 0.05; ** - p < 0.01; *** - p < 0.001. Individual data values in the Additional Table 3.

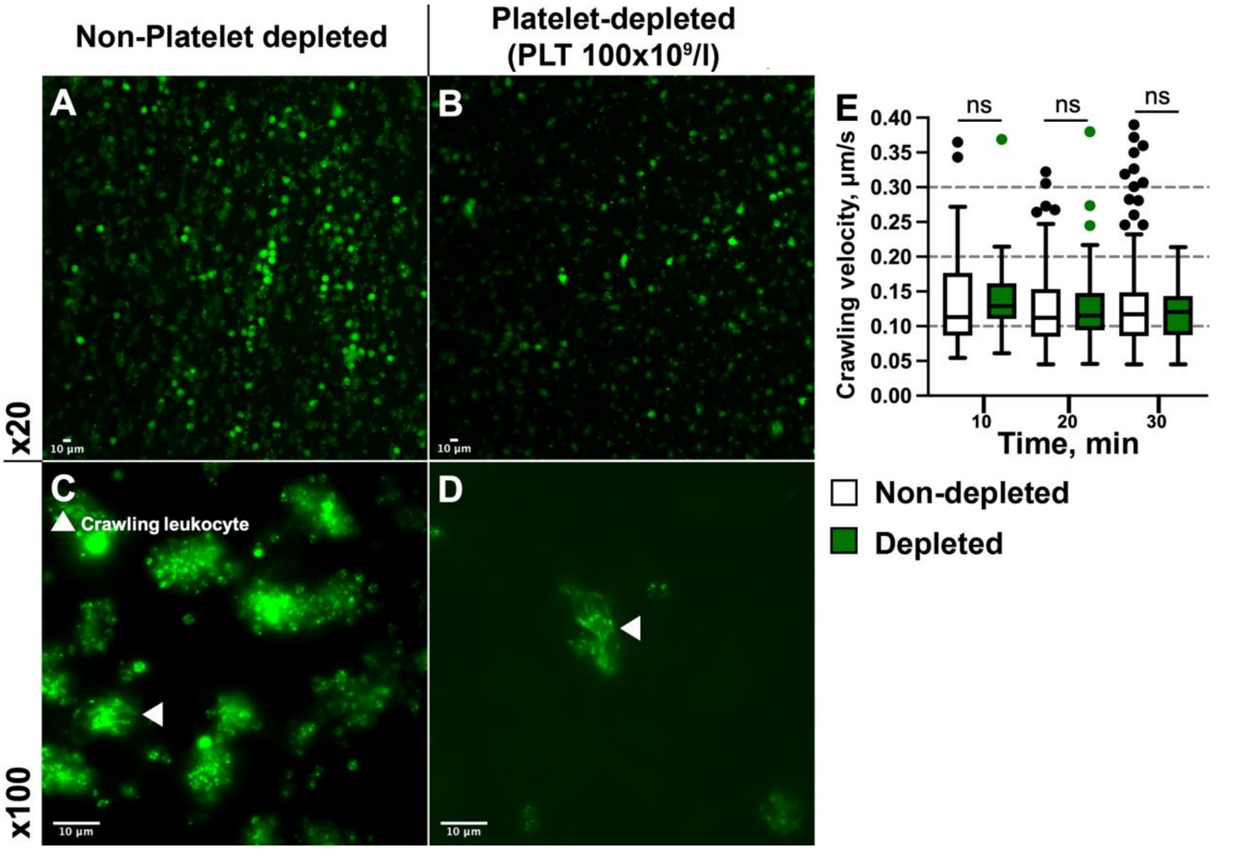

Figure S8. Impact of platelet depletion on granulocyte crawling velocity. A-D Typical FOVs platelet thrombi and crawling granulocytes of non-platelet-depleted (A,C) and platelet depleted (B,D) blood at 20x (A,B) and 100x (C,D) magnification. E – Comparison of the average granulocytes' crawling velocities in the non-platelet-depleted (white) and platelet-depleted (green). Statistical significance was calculated using Mann-Whitney Criteria. N = 5 healthy donors. Individual data values in the Additional Table 3.

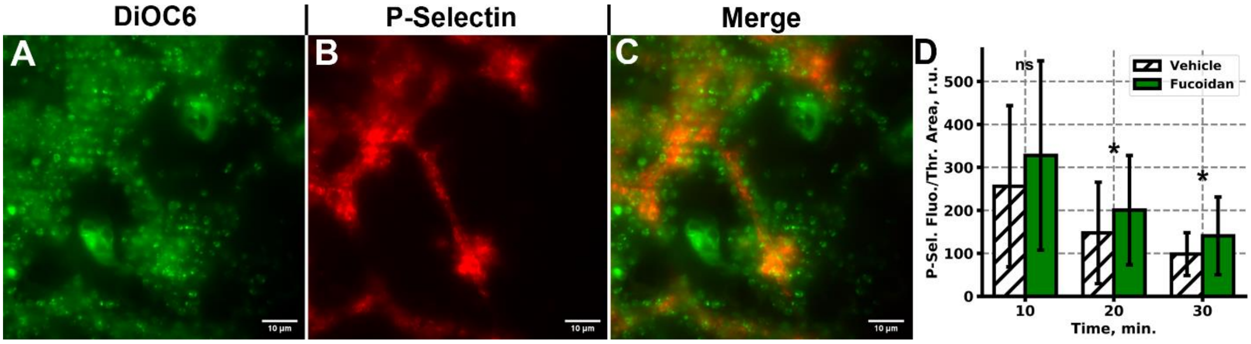

Figure S9. Fucoidan impact on platelet P-selectin exposure in growing thrombi. (A-C) Whole blood was dyed with DiOC6 and anti CD62p (P-Selectin) antibody (B). D - P-Selectin fluorescence was calculated in absolute units and then normalized on thrombus area. N = 5 healthy donors. Statistical significance was calculated based on Mann-Whitney criteria. * - p < 0.05 (https://doi.org/10.6084/m9.figshare.18603638). Individual data values in the Additional Table 3.

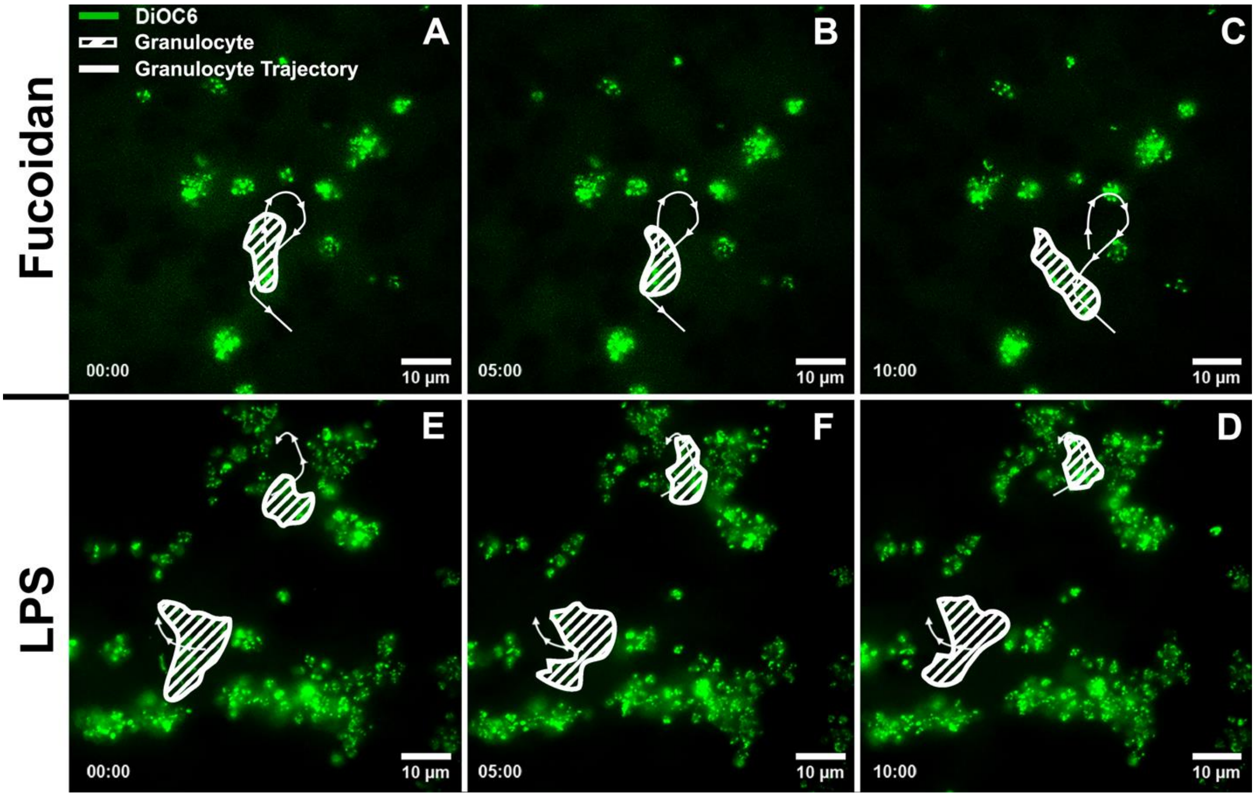

Figure S10. Granulocyte movement around thrombi. Pre-incubated with leukocyte activators anticoagulated whole blood. Images presented were captured on the 15th minute of perfusion. (A-D) Stained with DiOC6 (green) granulocyte and platelet cell membranes; granulocyte movement trajectories are shown with white arrows. Indices indicate the flow of time; (A-C) pre-incubation with pre-incubation with fucoidan (100 µg/ml); (E-D) pre- incubation with LPS (10 µg/ml) (https://doi.org/10.6084/m9.figshare.18357611.v1).

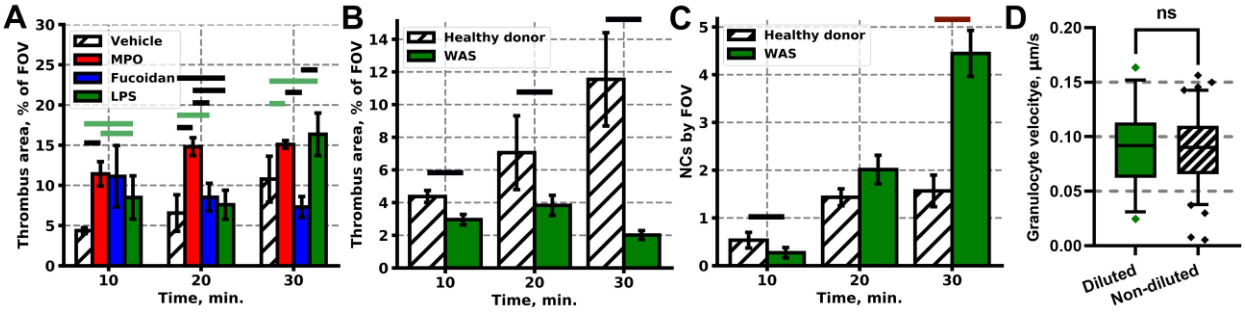

Figure S11. Thrombus area and percentage of highly activated granulocytes in the presence of leukocyte activators and in patients with WAS. (A) - Changes in thrombus area with the flow of time under the influence of blood pre-incubation with leukocytes activators; (B) - Changes in thrombus area with the flow of time in healthy pediatric donors and patients with WAS; (C) - percentage of highly spread leukocytes in patients with WAS is significantly higher from the start of the experiment up to 70% on the 20th minute. (D) Comparison between granulocyte velocity in the partially depleted and supplemented by Tyrode's plasma (Diluted) and normal whole blood (Non-Dilued). Individual data values in the Additional Table 3. Statistical significance was calculated with Mann-Whitney test, green lines indicate $p < 0.05$, red lines indicate $p < 0.01$, black lines indicate $p < 0.001$.

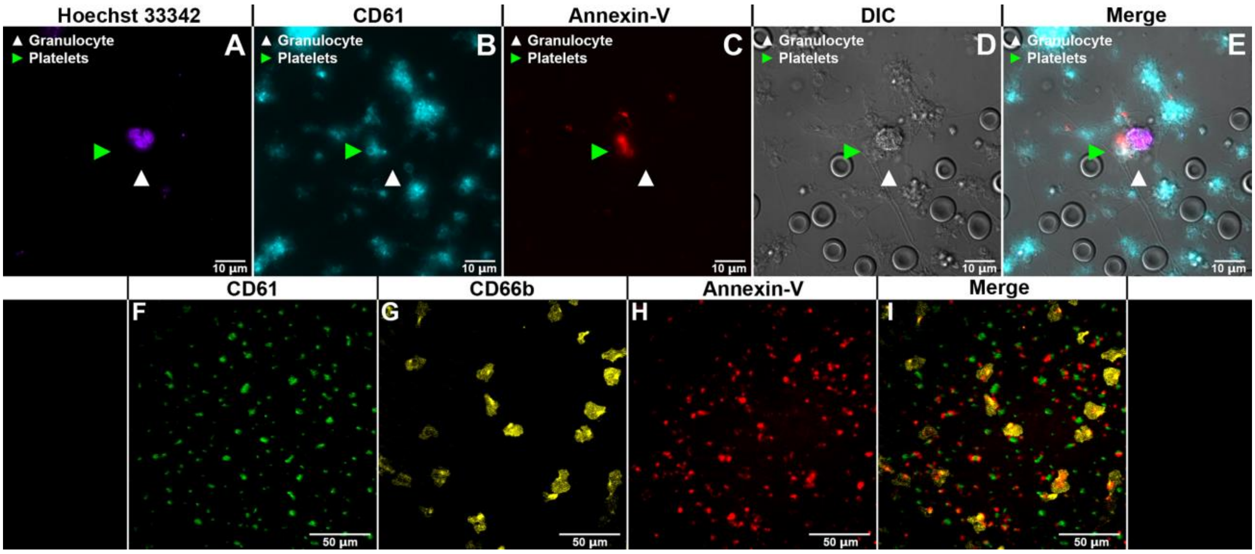

Figure S12. Crawling granulocytes bear Annexin-V positive platelets. (A-E) Whole blood was loaded with Hoechst 33342 for NC visualization (A), anti-CD61 for platelet visualization (B) and Annexin-V for the visualization of PS-positive (procoagulant, Annexin-V positive) platelets (https://doi.org/10.6084/m9.figshare.18419126.v1). (F-I) Whole blood was loaded with CD61 for platelet visualization (F), CD66b for granulocyte identification (G) and Annexin-V for phosphatidylserine positive cells identification (H). Representative data out of N=5 healthy donors (https://doi.org/10.6084/m9.figshare.18419285.v1).

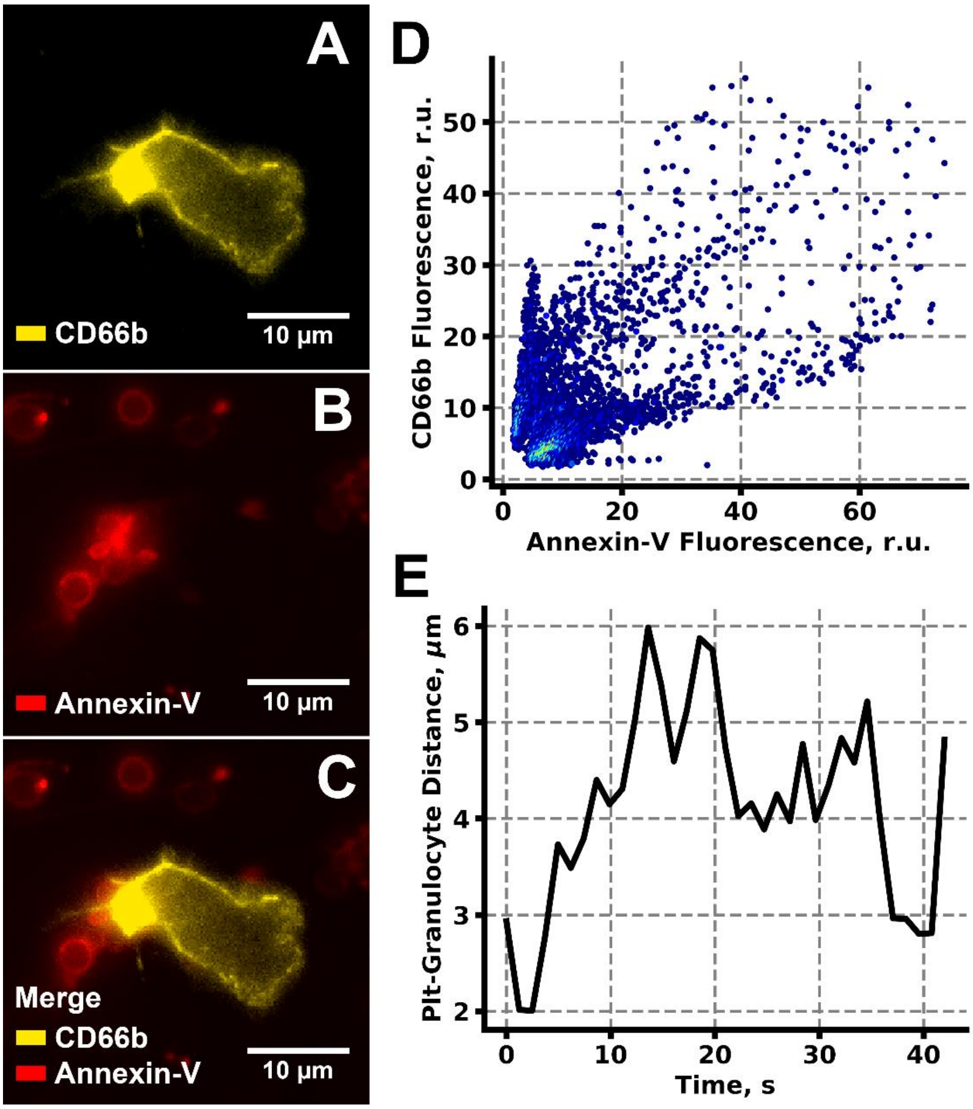

Figure S13. (A-C) CD66b (A) and Annexin (B) staining colocalized (C) in a manner suggesting that NCs bear Annexin-V positive platelets. (D) Scatterplot of the Annexin-V and CD66b fluorescence intensity correlation from (C). (E) Distance between the crawling neutrophil and Annexin-V positive platelet from Fig. 3J-L (https://doi.org/10.6084/m9.figshare.18610412).

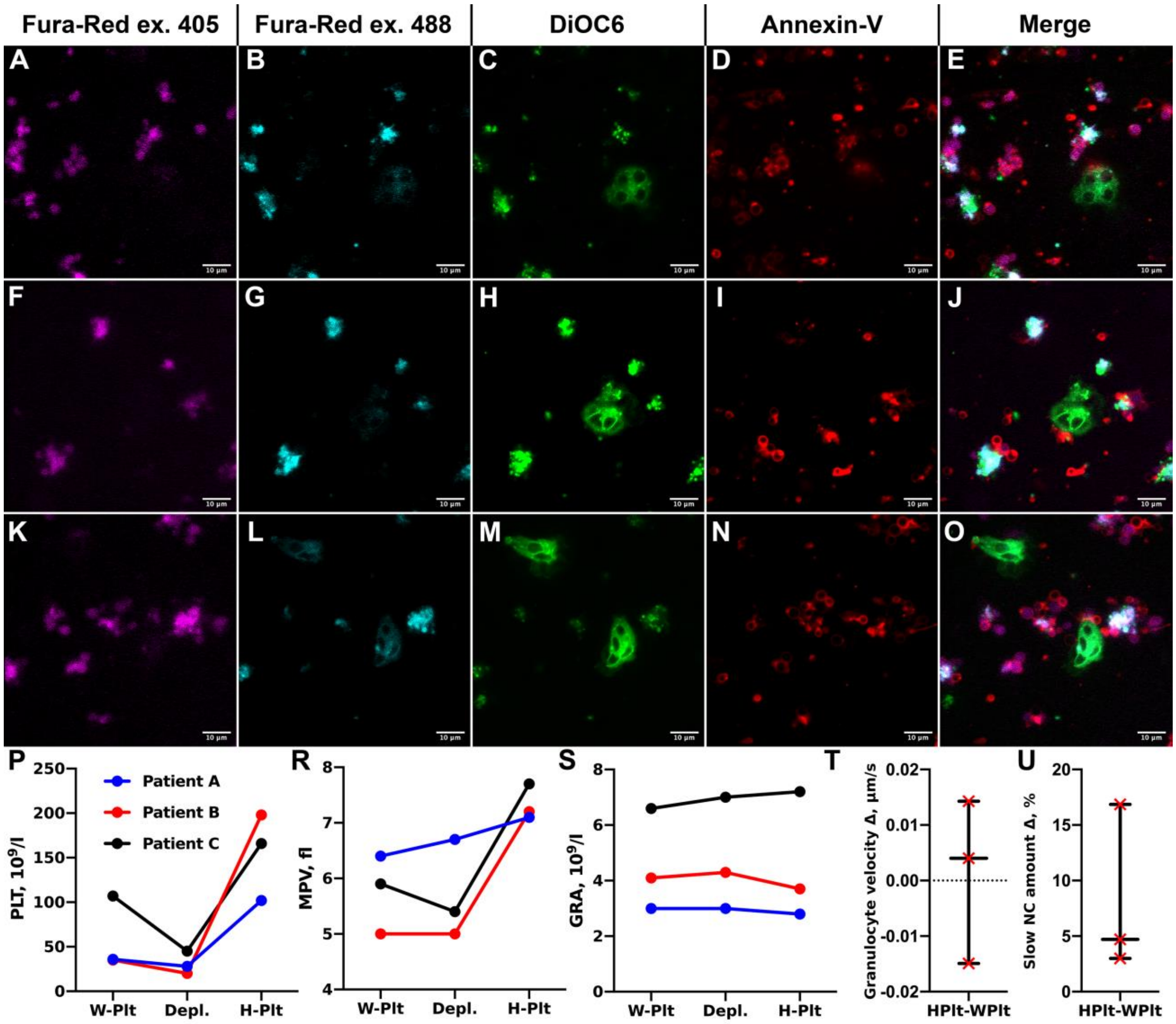

Figure S14. Assay of the granulocyte crawling in the blood of WAS patients in the presence of healthy donor platelets. (A-O) Typical fields of view of the WAS patient blood upon substitution of native WAS platelets by Fura-Red loaded healthy donor platelets: A,F,K – non bound to calcium Fura-Red; B,G,L – bound to calcium Fura-Red; C,D,M – DiOC6; D,I,N – Annexin-V; E,J,O – Merge (https://doi.org/10.6084/m9.figshare.18420278.v1). (P-S) – PLT (P), MPV (R) and GRA (S) of the patients in the native state (W-Plt), upon platelet depletion (Depl.) and after addition of healthy donor platelets (H-Plt). (T,U) – Difference between the average granulocyte velocity (T) and slow NC number (U) in the presence of healthy donor platelets (HPlt) and WAS native platelets (WPlt). N=3 WAS patients (https://doi.org/10.6084/m9.figshare.18358985.v1). Individual data values in the Additional Table 3.
